# Different subtypes of nonthyroidal illness syndrome on the prognosis of septic patients: a two-centered retrospective cohort study

**DOI:** 10.3389/fendo.2023.1227530

**Published:** 2023-09-08

**Authors:** Ning Ning, Juan Li, Wenwu Sun, Chaoping Ma, Jiaoyan Li, Huiqiu Sheng, Ying Chen, Bing Zhao, Jiyuan Zhang, Jiyue Zhu, Chengjin Gao, Enqiang Mao

**Affiliations:** ^1^ Departments of Emergency, Ruijin Hospital, School of Medicine, Shanghai Jiao Tong University, Shanghai, China; ^2^ Department of Emergency, Xinhua Hospital, Shanghai Jiao Tong University School of Medicine, Shanghai, China; ^3^ Department of General Surgery, Xinhua Hospital, Shanghai Jiao Tong University School of Medicine, Shanghai, China

**Keywords:** sepsis, nonthyroidal illness syndrome, low T3 syndrome, respiratory function, mortality

## Abstract

**Background:**

Nonthyroidal illness syndrome (NTIS) is a common endocrine dysfunction predicting unfavorable outcomes in critical illness. The objective of the study is to evaluate the association between different NTIS subtypes with outcomes in septic patients.

**Methods:**

Septic patients in two Chinese academic centers from October 2012 and October 2022 are enrolled in analysis. Multivariable regressions are used to assess associations between NTIS and outcomes. Outcomes include in-hospital mortality, length of stay in hospital (LOS), non-invasive ventilation failure and weaning failure. Patients with NTIS are categorized into 4 types according to the different levels of FT4 and TSH. The association between different NTIS subtypes and mortality are further analyzed. Survival curve is plotted using the Kaplan–Meier method.

**Results:**

After screening, a total of 1226 septic patients with complete thyroid hormones result are eventually enrolled. Among them, 520 (42.4%) patients are diagnosed as NTIS. In multivariable regression analysis, NTIS is independently associated with increased 30-days mortality (OR=1.759, CI 1.009-3.104, p=0.047), but has no association with 60-days mortality (OR=1.524, CI 0.893-2.618, p=0.123), 90-days mortality (OR=1.411, CI 0.831-2.408, p=0.203), LOS, non-invasive ventilation failure or weaning failure. In NTIS subtypes, NTIS patients with low FT3 and TSH levels, regardless of the FT4 values, have significantly higher mortality than euthyroid patients (30-days mortality, OR= 6.488, CI 1.546-27.808, p=0.01; 60-days mortality, OR=3.973, CI 1.006-15.579, p=0.046; 90-days mortality, OR=3.849, CI 0.977-15.088, p=0.051). This result is consistent in patients with low FT3 and FT4 levels, regardless of the TSH values (30-days mortality, OR=3.349, CI 1.402-7.957, p=0.006; 60-days mortality, OR= 2.594, CI 1.122-5.930, p=0.024; 90-days mortality, OR=2.55, CI 1.110-5.804, p=0.025). There is no survival difference between NTIS patients with low FT3 only and euthyroid patients. Survival plot shows the worst prognosis is in NTIS patients with low FT3, FT4 and TSH level.

**Conclusions:**

NTIS is frequent in sepsis. A reduction of FT3 together with FT4 or TSH, but not FT3 only, is associated with an increased risk of mortality.

## Background

Sepsis is an infection induced life-threatening syndrome, which impacts millions of people around the world each year (1). Because of the aging populations, the reported incidence of sepsis is still increasing (2). For the better prognosis of septic patients, the treatment and management methods are still evolving.

Neuroendocrine systems dysfunction could contribute to sepsis-induced organ failure (3). Nonthyroidal illness syndrome (NTIS), also called “low T_3_ syndrome” or “euthyroid sick syndrome”, is the most common endocrine dysfunction in critical illness (4). The most obvious laboratory finding in NTIS is a dropped triiodothyronine (T_3_) with low or normal levels of thyroxine (T_4_) or thyroid stimulating hormone (TSH) in the absence of previous thyroidal diseases (5). The mechanism of NTIS in sepsis is not fully understood yet. One explanation is the theory of the malfunction of hypothalamic-pituitary-thyroid axis by inflammatory cytokine or some drugs (6, 7). This theory is supported by the evidence that TSH level is decreased in a part of critically ill patients. Another explanation is the theory of impaired T4 metabolism in peripheral tissues. This theory is supported by the evidence that the reverse T3 (rT3), an inactive peripheral metabolite of T4, is increased in critically ill patients (8).

Previous studies have proved NTIS is associated with an unfavorable outcomes in septic patients (9). However, the pathophysiological significance of NTIS in sepsis could not be confirmed. The thyroid hormones change in critically ill patients has been regarded as similar as the condition of starvation, in which low T3 level is seen as the part of an adaptive metabolic response in an attempt to ameliorate the stress by lowering the metabolic activity (10). But this view is challenged by some suggesting that NTIS represents a maladaptive response to illness that requires correction. The question of whether or not septic patients will gain benefit from treating NTIS could not be answered yet. This question could only be answered in large randomized control trials (RCT). Some RCTs have been published not specifically for septic patients. However, the result is still inclusive (11). Surely, it should be pointed out that normalizing thyroid hormone concentrations is not necessarily equal to normalizing local thyroid hormone action, especially in critical illness (12). Astonishingly, no RCT result has been published to assess the treatment effects in septic patients with NTIS.

The objective of the study is therefore to identify subgroups of septic patients who might benefit from endocrine treatment in future RCTs.

## Methods

### Participants

This retrospective study was conducted in emergency intensive care unit (ICU), respiratory ICU and general ICU of Ruijin Hospital, Shanghai Jiaotong University School of Medicine, an academic 2100 beds hospital; and Xinhua Hospital, Shanghai Jiaotong University School of Medicine, an academic 2000 beds hospital. The protocol was approved by the institutional ethics board of each hospital (RJ-2022-312, XHEC-D-2023-074). The informed consent was waived because of the non-interventional, retrospective design of the study. Data analysis was performed in accordance with the 1964 Helsinki Declaration and its later amendments.

Consecutive adult patients with a diagnosis of sepsis between September 2015 and May 2022 (Ruijin Hospital, Shanghai Jiaotong University School of Medicine), and between October 2012 and October 2022 (Xinhua Hospital, Shanghai Jiaotong University School of Medicine) were screened. All patients were treated according to our local protocol, which included administering antibiotics as early as possible and initiating fluid resuscitation with crystalloids in addition to source control. Patient exclusion criteria included (1): without thyroid hormones test within 24h after admission (2) less than 48 hours from admission to discharge or death; (3) history of malignancy or autoimmune diseases; (4) history of thyroidal or pituitary diseases; (5) using drug that interfere with the hypothalamic-pituitary-thyroid axis before thyroid hormones test, such as contrast agents, amiodarone, thyroxine, thyreostatic agents, dopamine, lithium, antidepressants, and glucocorticoids.

### Data collection

The clinical variables are extracted from the paper-based or electronic medical records for each patient. Baseline demographic information includes age, sex, comorbidities (hypertension, diabetes and chronic kidney diseases). Infection sites are classified into respiratory system, gastrointestinal tract, urogenital tract, and others. Laboratory indicators were collected within 24 hours after admission included white blood cell count, alanine aminotransferase, total bilirubin, creatinine, lactate, C-reactive protein, procalcitonin and platelets count. Thyroid function (including TT3, FT3, TT4, FT_4_, TSH and rT3), Acute Physiology and Chronic Health Evaluation II scores (APACHE II) and Sequential Organ Failure Assessment (SOFA) scores were collected within 24 hours after admission. The use of non-invasive ventilation, invasive ventilation, and vasopressors were reported as well. Outcomes include in-hospital mortality, length of stay in hospital (LOS), non-invasive ventilation failure and weaning failure. Non-invasive ventilation failure is defined as oxygenation could not be maintained by non-invasive ventilation, and invasive ventilation with intubation or tracheotomy should be used. Weaning failure is defined as oxygenation could not be maintained after spontaneous breathing trial, and tracheostomy should be used; or patients suffered second intubation within 24h after extubation.

### Definition

The diagnosis of sepsis is defined as SOFA score >= 2 points consequent to the infection (Sepsis-3) (1). NTIS in this study is defined as a low serum FT3 level (<2.63pmol/L) without an elevated TSH level (< 4.94μIU/mL). The normal range of serum thyroid hormones in this study: FT3, 2.63–5.7pmol/L; FT4, 9.01–19.04pmol/L; TT3, 0.89–2.44nmol/L; TT4, 62.67–150.84nmol/L; rT3, 31-95ng/dl; and TSH, 0.35–4.94μIU/mL. Patients with NTIS are categorized into 4 types: type 1, low FT3, normal FT4 and TSH; type 2, low FT3, normal FT4, and low TSH; type 3, low FT3, low FT4, and normal TSH; type 4, low FT3, low FT4 and TSH.

### Data statistics

Categorical data will be described with frequency or ratio. Continuous variables will be described using median and interquartile range. Categorical variables will be compared using χ2 test or Fisher exact test. Continuous variables will be compared using the t-test for normally distributed variables or Wilcoxon rank-sum test for non-normally distributed variables. We implement multiple predictive mean matching imputations using chained equation and created 5 independent datasets to compute missing data. Variables with missing data more than 20% are deleted.

Multivariable logistic regression is used to assess associations between 30-days mortality, 60-days mortality, 90-days mortality and NTIS. Multivariable linear regression analysis is used to assess association between LOS and NTIS. Effect sizes are reported as odds ratios (OR) and β estimates, respectively. Baseline information include age, sex, comorbidities, infection sites are adjusted. Moreover, total bilirubin represents liver function; creatinine represents renal function; lactate represents oxygen debt; C-reactive protein represents overall inflammation; procalcitonin represents infection severity; platelets count represents coagulation function; APACHE II score, use of non-invasive ventilation, invasive ventilation, and vasopressors are known associated with illness severity. These variables are also adjusted in the models. A subgroup of patients underwent non-invasive ventilation is selected to estimate the effect of NTIS on the incidence of non-invasive ventilation failure. A subgroup of patients underwent invasive ventilation is selected to estimate the effect of NTIS on the incidence of weaning failure. Multivariable logistic regression is used and OR is reported. Considering the limited number of patients in subgroups, only variables with significant difference in univariable logistic regression (p<0.05) are adjusted. Survival curve is plotted using the Kaplan–Meier method. All statistical analyses are performed using R software (version 4.2.1). A two-sided significance level less than 0.05 are defined as statistical significance.

## Results

**Figure 1:**
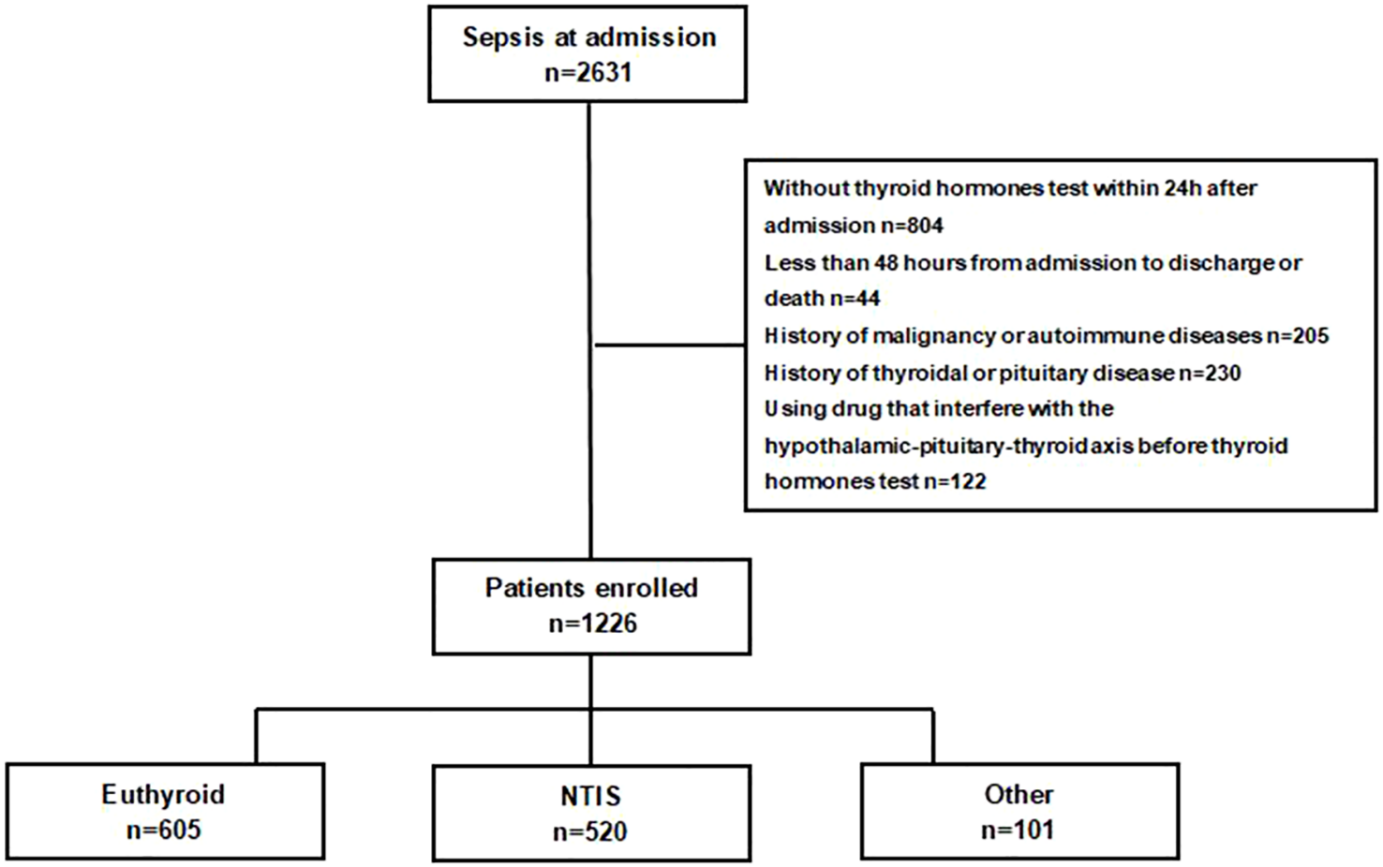
Screening flowchart. NTIS, nonthyroidal illness syndrome.

A total of 1226 septic patients are eventually enrolled in our study. Among them, 605 (49.3%) patients are euthyroid with normal serum level of FT3, FT4 and TSH; 520 (42.4%) patients are diagnosed as NTIS with a low serum level of FT3 without elevated TSH. The other 101 (8.3%) patients are diagnosed with other thyroid hormones abnormality, but not included in the following analysis. The selection flowchart is shown in **Figure 1**.

### Patients’ characteristics

A total of 1125 septic patients with euthyroid or NTIS are included in analysis. Patients with NTIS are older (median 72 vs 70, p =0.01), with fewer male (56.3% vs 65.3%, p=0.002), and have higher percentage of diabetes (42.1% vs 31.6%, p<0.001) and chronic kidney diseases (27.3% vs 17.5%, p<0.001). Most of the laboratory indicators on admission are worse in patients with NTIS, except for alanine aminotransferase (median 28 vs 28, p=0.238) and lactate (median 1.9 vs 1.7, p=0.404). As expected, the NTIS is associated with the severity of disease. APACHEII score (median 14 vs 10, p<0.001) and SOFA score (median 6 vs 3, p<0.001) are much higher in patients with NTIS. Patients with NTIS are more likely to receive non-invasive ventilation (24.6% vs 12.6%, p<0.001), invasive ventilation (21.5% vs 6.4%, p<0.001), and use vasopressors (34.6% vs 12.9%, p<0.001) than euthyroid patients (**Table 1**).

**Table 1:** Baseline characteristics between patients with NTIS and without NTIS.

Characteristics	Total (n=1125)	With NTIS (n=520)	Without NTIS (n=605)	p value
**Age, years**	71 (62-81)	72 (64-82)	70 (61-80)	0.01*
**Sex (male%)**	688 (61.2)	293 (56.3)	395 (65.3)	0.002*
Comorbidities				
** Hypertention**	587 (52.2)	274 (52.7)	313 (51.7)	0.749
** Diabetes**	410 (36.4)	219 (42.1)	191 (31.6)	<0.001*
** Chronic kidney diseases**	248 (22.0)	142 (27.3)	106 (17.5)	<0.001*
Infection sites				
** Respiratory system**	721 (64.1)	362 (69.6)	359 (59.3)	<0.001*
** Gastrointestinal tract**	294 (26.1)	123 (23.7)	171 (28.3)	0.079
** Urogenital tract**	283 (25.2)	116 (22.3)	167 (27.6)	0.041*
** Others**	141 (12.5)	78 (15.0)	63 (10.4)	0.021*
Laboratory indicators				
** White blood cell count, ×109/L**	10.2 (6.8-14.9)	11.8 (8.2-17.0)	8.9 (5.9-13.0)	<0.001*
** Alanine aminotransferase, IU/L**	28 (16-59)	28 (16-62)	28 (16-55)	0.238
** Total bilirubin, μmol/L**	15.2 (10.0-23.9)	16.6 (10.2-26.3)	14.2 (9.9-21.3)	0.001*
** Creatinine, μmol/L**	88 (66-146)	108 (71-214)	79 (63-114)	<0.001*
** Lactate, mmol/L**	1.8 (1.3-2.4)	1.9 (1.4-2.7)	1.7 (1.3-2.3)	0.404
** C-reactive protein, mg/L**	114 (47-160)	146 (74-172)	89 (32-160)	<0.001*
** Procalcitonin, ng/mL**	1.9 (0.3-14.8)	3.2 (0.7-22.0)	1.0 (0.2-9.5)	<0.001*
** Platelets count, ×109/L**	149 (97-206)	141 (84-206)	155 (107-205)	0.008*
**APACHEII score**	12 (8-17)	14 (10-20)	10 (7-14)	<0.001*
**SOFA score**	4 (3-7)	6 (3-8)	3 (2-5)	<0.001*
**Non-invasive ventilation**	204 (18.1)	128 (24.6)	76 (12.6)	<0.001*
**Invasive ventilation**	151 (13.4)	112 (21.5)	39 (6.4)	<0.001*
**Vasopressors**	258 (22.9)	180 (34.6)	78 (12.9)	<0.001*

Data are presented as median (interquartile range), or number (percentage).

NTIS, nonthyroidal illness syndrome; APACHE II, Acute Physiology and Chronic Health Evaluation II; SOFA, Sequential Organ Failure Assessment.

*p < 0.05.

### Associations between NTIS and outcomes

In multivariable logistic regression, after adjustment for confounders, NTIS is independently associated with increased 30-days mortality (OR=1.759, CI 1.009-3.104, p=0.047), but has no association with 60-days mortality (OR=1.524, CI 0.893-2.618, p=0.123) and 90-days mortality (OR=1.411, CI 0.831-2.408, p=0.203). In multivariable linear regression analysis, after adjustment for confounders, there is no association between NTIS and LOS (β estimate=-1.973, CI -5.485 to 1.537, p=0.270). A subgroup of patients underwent non-invasive ventilation (n=204). The incidence of non-invasive ventilation failure is 34.3% (n=70). Baseline characteristics between patients with or without non-invasive ventilation failure are shown in **Supplemental Table 1**. In multivariable logistic regression, after adjustment for age, sex, C-reactive protein, APACHEII score and use of vasopressors, there is no association between NTIS and non-invasive ventilation failure (OR=1.774, CI 0.840-3.828, p=0.136). A subgroup of patients underwent invasive ventilation (n=151). The incidence of weaning failure is 35.7% (n=54). Baseline characteristics between patients with or without weaning failure are shown in **Supplemental Table 2**. In multivariable logistic regression, after adjustment for age, sex, chronic kidney diseases and APACHEII score, there is no association between patients NTIS and weaning failure (OR=1.437, CI 0.624-3.453, p=0.402). The associations are shown in **Table 2**.

**Table 2:** Multivariable Analyses of associations between NTIS and outcomes.

	OR	CI	p value
In-hospital mortality			
** 30-days mortality**	1.759	1.009-3.104	0.047*
** 60-days mortality**	1.524	0.893-2.618	0.123
** 90-days mortality**	1.411	0.831-2.408	0.203
**Non-invasive ventilation failure%**	1.774	0.840-3.828	0.136
**Weaning failure#**	1.437	0.624-3.453	0.402
	β estimate	CI	p value
**Length of stay in hospital**	-1.973	-5.485 to 1.537	0.270

NTIS, nonthyroidal illness syndrome; OR, odds ratio; CI, confidence interval;

Models adjusted for age, sex, all of comorbidities, all of infection sites, total bilirubin, creatinine, lactate, C-reactive protein, procalcitonin, platelets count, APACHEII score, non-invasive ventilation,invasive ventilation and vasopressors.

% adjusted for age, sex, C-reactive protein, APACHEII score and vasopressors.

# adjusted for age, sex, chronic kidney diseases and APACHEII score.

*p < 0.05.

### Characteristics and outcomes of patients with different NTIS types

**Figure 2:**
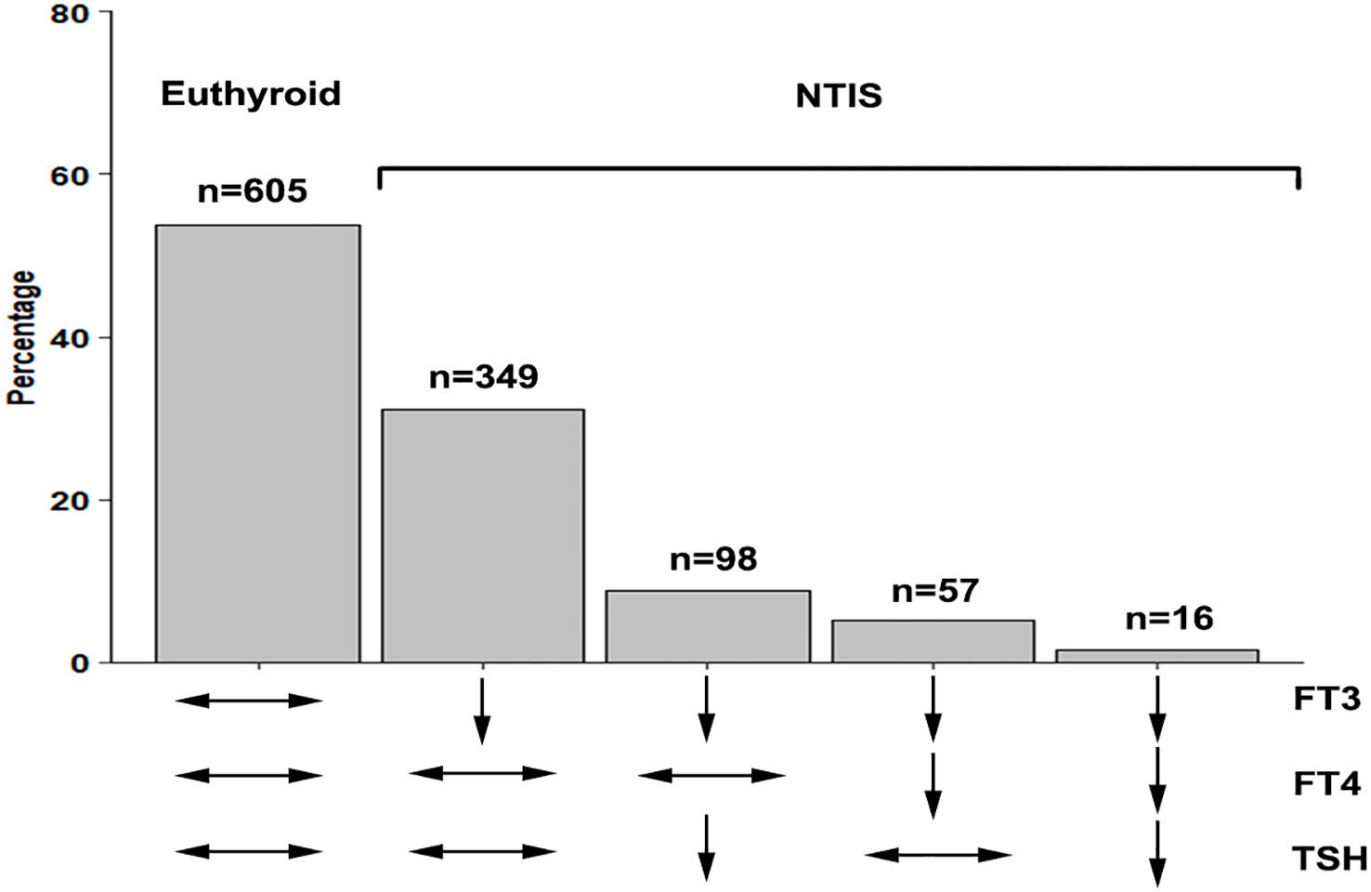
The patient number of different types NTIS. NTIS, nonthyroidal illness syndrome; FT3, free triiodothyronine; FT4, free thyroxine; TSH, thyroid stimulating hormone.

**Figure 3:**
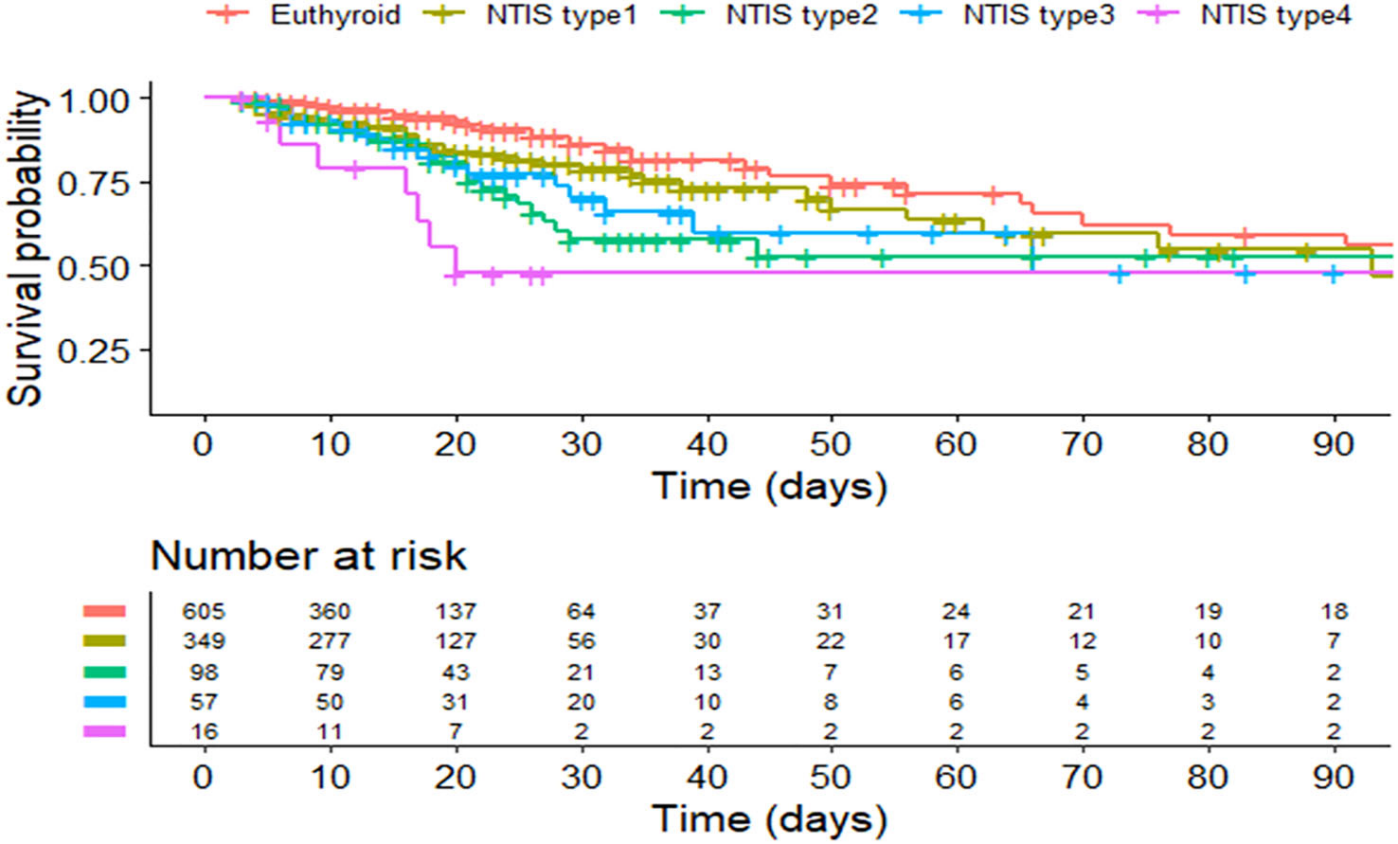
Kaplan–Meier curve of the euthyroid and different NTIS types. NITS, nonthyroidal illness syndrome.

A total of 520 patients with NTIS are categorized into 4 types according to the different levels of FT4 and TSH: 349 (67.1%) patients are classified into type 1; 98(18.8%) patients are classified into type 2; 57 (10.9%) patients are classified into type 3; and 16 (3.2%) patients are classified into type 4. The patient number and thyroid hormones characteristics of different types NTIS are shown in **Table 3**; **Figure 2**. **Figure 3** compares the cumulative survival rates between the euthyroid and different NTIS types. As shown in the survival curve, patients with NTIS type 4 (low FT3, FT4 and TSH) have the highest mortality rate compared with euthyroid or other types NTIS. Considering the low percentage of NTIS type 3and NTIS type 4 in the total patients, different NTIS types are grouped for further analysis. NTIS group 1: low FT3 and TSH levels, regardless of the FT4 values (NTIS type 2+NTIS type 4). NTIS group 2: low FT3 and FT4 levels, regardless of the TSH values (NTIS type 3+NTIS type 4). As shown in **Table 4**, patients with low FT3 and TSH levels, regardless of the FT4 values, have significantly higher incidence of mortality than euthyroid patients (30-days mortality, OR= 6.488, CI 1.546-27.808, p=0.01; 60-days mortality, OR=3.973, CI 1.006-15.579, p=0.046; 90-days mortality, OR=3.849, CI 0.977-15.088, p=0.051). But there is no significant difference between patients with low FT3 only and enthyroid (30-days mortality, OR=1.489, CI 0.788-2.827, p=0.219; 60-days mortality, OR=1.399, CI 0.762-2.570, p=0.276; 90-days mortality, OR=1.335, CI 0.730-2.241, p=0.345). Similarly, patients with low FT3 and FT4 levels, regardless of the TSH values, have significantly higher incidence of mortality than euthyroid patients (30-days mortality, OR=3.349, CI 1.402-7.957, p=0.006; 60-days mortality, OR= 2.594, CI 1.122-5.930, p=0.024; 90-days mortality, OR=2.55, CI 1.110-5.804, p=0.025). But there is no significant difference between patients with low FT3 only and enthyroid (30-days mortality, OR=1.564, CI 0.842-2.925, p=0.157; 60-days mortality, OR=1.376, CI 0.760-2.494, p=0.29; 90-days mortality, OR=1.282, CI 0.710-2.314, p=0.407).

**Table 3:** The thyroid hormones characteristics of different types NTIS.

Thyroid function	Euthyroid (n=605)	NTIS type1 (n=349)	NTIS type2 (n=98)	NTIS type3 (n=57)	NTIS type4 (n=16)
**FT3, pmol/L**	3.0 (2.7-3.4)	1.9 (1.6-2.2)	2.0 (1.7-2.1)	1.5 (1.5-1.8)	1.6 (1.5-1.9)
**FT4, pmol/L**	14.2 (12.7-15.9)	12.3 (10.9-13.8)	12.9 (11.4-14.6)	8.2 (7.4-8.6)	7.8 (7.2-8.4)
**TSH, μIU/mL**	1.4 (0.8-2.2)	1.0 (0.6-1.6)	0.2 (0.1-0.3)	1.1 (0.6-1.9)	0.2 (0.1-0.2)
**TT3, nmol/L**	1.0 (0.8-1.2)	0.6 (0.5-0.7)	0.6 (0.4-0.7)	0.5 (0.4-0.6)	0.4 (0.3-0.5)
**TT4, nmol/L**	91.8 (75.9-110.4)	68.6 (56.9-79.7)	67.0 (52.6-84.5)	39.8 (31.9-47.2)	34.6 (31.9-41.1)
**rT3, ng/dl**	0.5 (0.4-69.6)	0.5 (0.3-107.2)	0.5 (0.3-96.6)	46.9 (2.9-96.7)	83.1 (0.5-101.7)

Data are presented as median (interquartile range).

NTIS, nonthyroidal illness syndrome; FT3, free triiodothyronine; FT4, free thyroxine; TSH, thyroid stimulating hormone; TT3, total triiodothyronine; TT4, total thyroxine; rT3, reverse triiodothyronine.

**Table 4:** Multivariable logistic analysis of associations between different NTIS types and mortality.

	Euthyroid	NTIS type 1	NTIS group 1
**30-days mortality**	1	1.489	6.488
	(ref)	0.788-2.827	1.546-27.808
		0.219	0.01*
**60-days mortality**	1	1.399	3.973
	(ref)	0.762-2.570	1.006-15.579
		0.276	0.046*
**90-days mortality**	1	1.335	3.849
	(ref)	0.730-2.241	0.977-15.088
		0.345	0.051
**30-days mortality**	1	1.564	3.349
	(ref)	0.842-2.925	1.402-7.957
		0.157	0.006*
**60-days mortality**	1	1.376	2.594
	(ref)	0.760-2.494	1.122-5.930
		0.29	0.024*
**90-days mortality**	1	1.282	2.55
	(ref)	0.710-2.314	1.110-5.804
		0.407	0.025*

NTIS, nonthyroidal illness syndrome.

NTIS type 1: low FT3 only; NTIS group 1:low FT3 and TSH levels, regardless of the FT4 values; NTIS group 2:low FT3 and FT4 levels, regardless of the TSH values.

Models adjusted for age, sex, all of comorbidities, all of infection sites, total bilirubin, creatinine, lactate, C-reactive protein, procalcitonin, platelets count, APACHEII score, non-invasive ventilation,invasive ventilation and vasopressors.

*p<0.05

## Discussion

Since first described in 1970s, NTIS has been viewed as a predictor of unfavorable outcomes in critical illness (4). However, there is a lack of RCT evaluating treatment effect on the NTIS in sepsis. One important reason may be that there are disappointing results in the trials of other critical illness. In previous studies, cardiac diseases and cardiac surgery was the interesting field of investigation. But the results showed that, even though the short duration thyroid hormones treatment could increase the cardiac index, there is no improvement on the post-operative mortality (13). In another meta-analysis of congenital heart surgery in children, thyroid hormone replacement could ameliorate inotrope score, but not effective on mechanical ventilation duration, LOS and post-operative mortality (14). However, it must be pointed out that, the conclusion could not be extrapolated to sepsis directly due to the small sample size and different etiologies of these trials.

It is very crucial to select valid endpoints to assess the potential benefit of treatment. Selection of non-appropriate endpoints could result in failure of the trial. Mortality is the most concerned endpoint in clinical trials on sepsis. Todd et al. reported that decreased T3 level at baseline is associated with mortality in 231 surgical septic patients (15). In Wang’s study, it was also reported that decreased serum T4 level can be a predictive marker for in-hospital mortality (16). In our study, the short term 30 days in-hospital mortality is independently associated with NTIS. This conclusion is consistent with previous reports. However, multiple organs and systems could be involved in sepsis. There is lack of evidence regarding the final targets of the thyroid hormones in sepsis. A large part of septic patients suffered respiratory failure, which is a main cause of mortality. Diaphragm and muscle weakness could be induced by sepsis and is correlated with poor outcomes (17, 18). Thyroid hormone is important to normal skeletal muscle function. So it was speculated that the weakness of muscular strength secondary to NTIS increases the risk of mortality in septic patients. We found that patients with NTIS have an increased need for non-invasive ventilation and invasive ventilation, which in addition to signifying a worse clinical condition, may also be an additional indication of skeletal muscle weakness, including the diaphragm. However, NTIS has no association with non-invasive ventilation failure and weaning failure in this study. In Flavia’s research, impaired mitochondrial function of diaphragm muscle could be observed and thyroid hormone treatment could improve mitochondrial parameters on the septic mouse model (19). But the result could not be repeated in human study, that thyroid hormone treatment did not yield any benefit on respiratory muscle function in critically ill patients with NTIS (20).

One important finding of our study is that different NTIS subtypes could significantly impact the mortality in sepsis, which means thyroid hormones pattern heterogeneity should be considered in the patients diagnosed with NTIS. In our study, after excluding hypothalamic-pituitary-thyroid axis diseases, we found NTIS patients with low FT4 independent from the TSH levels, or low TSH independent from the FT4 levels had significantly higher mortality than euthyroid patients. But there is no survival difference between NTIS patients with low FT3 only and euthyroid patients. The worst prognosis is in the group of patients with low FT3, FT4 and TSH levels. Similar conclusion is suggested in another study on 247 critical illness patients (21). But in this study, it was concluded that only low FT3 and FT4 levels is an independent risk factor for mortality; low TSH level has no impact on the prognosis. This conclusion should be interpreted carefully because of the limited number of low TSH level patients in this study. The reduced FT4 or TSH indicates the suppressed hypothalamic-pituitary-thyroid axis response. In previous RCTs, intravenous or oral thyroid hormones substitutions were the choice of intervention (11). However, thyroid hormones substitutions may further suppress the hypothalamic-pituitary-thyroid axis. In animal sepsis models, NTIS can be overcomed by thyrotropin releasing hormone (TRH) administration, confirming that stimulation of the pituitary may restore the decreased serum thyroid hormone levels in sepsis (22). In protracted critical ill patients, administration of TRH and growth hormone-releasing peptide can reactivate blunted TSH secretion and restore plasma thyroid hormone levels (23). These studies provide convincing evidence that the hypothalamic-pituitary-thyroid axis is a possible treatment target in NTIS.

There are certain limitations in this study. Firstly, although the data in this study are prospectively collected, this remains a retrospective study, and missing data is inevitable. Even though we do our best to fill the missing value with multiple imputations, the bias still exists. Secondly, the thyroid hormone levels are changed dynamically during the sepsis process, longitudinal dynamic measurement of thyroid hormone levels is not available. Thirdly, although our study has a large sample size, it was performed on the Chinese population, and only two centers are included. Finally, the cause of the sepsis is not elucidated in this study. However, to our knowledge, this is the first study that comprehensively analyzes the association between different thyroid hormone patterns of NTIS and prognosis in sepsis. This study may provide information about stratification of septic patients and choice of treatment methods in future clinical trials.

## Conclusions

In summary, almost half of septic patients complicated with NTIS. Septic patients with NTIS have significantly higher 30 days in-hospital mortality. NTIS patients with low serum FT4 or TSH level have lower survival rate than euthyroid patients. There is no survival difference between NTIS patients with low FT3 only and euthyroid. The worst prognosis is in NTIS patients with low FT3, FT4 and TSH level. Different patterns of NTIS should be considered when enrolling participants or choosing treatment methods in future randomized trials about sepsis.

## Data availability statement

The raw data supporting the conclusions of this article will be made available by the authors, without undue reservation.

## Ethics statement

The studies involving humans were approved by the institutional ethics board of Ruijin Hospital, Shanghai Jiaotong University School of Medicine and Xinhua Hospital, Shanghai Jiaotong University School of Medicine. The studies were conducted in accordance with the local legislation and institutional requirements. The ethics committee/institutional review board waived the requirement of written informed consent for participation from the participants or the participants’ legal guardians/next of kin because The informed consent was waived because of the non-interventional, retrospective design of the study.bnh

## Author contributions

NN, Juan Li, WS and CM: original draft. WS: analysis. Jiaoyan Li, BZ, JZhang and JZhu: data collection. HS and YC: supervision. CG and EM: conceptualization. All authors read and approved the final manuscript. All authors contributed to the article and approved the submitted version.
