# Supplementary material for: Different subtypes of nonthyroidal illness syndrome on the prognosis of septic patients: a two-centered retrospective cohort study
Source: Front Endocrinol (Lausanne). 2023 Sep 8;14:1227530. doi: 10.3389/fendo.2023.1227530 (PMC10517721; doi:10.3389/fendo.2023.1227530)
Supplement: Supplementary file 1 [file Table_1.docx]

Supplemental Table 1. Baseline characteristics between NTIS and euthyroid patients in the non-invasive ventilation subgroup.

| **Characteristics** | **Total (n=204)** | **Without non-invasive ventilation failure (n=134)** | **With non-invasive ventilation failure (n=70)** | **p value** |
| --- | --- | --- | --- | --- |
| **Age, years** | 73(66-81) | 76(67-84) | 69(63-78) | 0.01* |
| **Sex (male%)** | 138(67.6) | 89(66.4) | 49(70.0) | 0.604 |
| **Comorbidities** |  |  |  |  |
| **Hypertention** | 122(59.8) | 82(61.2) | 40(57.1) | 0.575 |
| **Diabetes** | 77(37.7) | 53(39.6) | 24(34.3) | 0.461 |
| **Chronic kidney diseases** | 66(32.4) | 46(34.3) | 20(28.6) | 0.404 |
| **Infection sites** |  |  |  |  |
| **Respiratory system** | 186(91.2) | 121(90.3) | 65(92.9) | 0.725 |
| **Gastrointestinal tract** | 17(8.3) | 11(8.2) | 6(8.6) | 0.929 |
| **Urogenital tract** | 40(19.6) | 33(24.6) | 7(10.0) | 0.012* |
| **Others** | 5(2.5) | 3(2.2) | 2(2.9) | 1 |
| **Laboratory indicators** |  |  |  |  |
| **White blood cell, ×109/L** | 11.5(7.8-15.4) | 11.8(7.6-15.8) | 10.9(8.6-15.0) | 0.269 |
| **Alanine aminotransferase, IU/L** | 27(16-53) | 26(19-49) | 30(14-72) | 0.309 |
| **Total bilirubin, μmol/L** | 14.4(10.3-22.3) | 15.2(10.6-22.1) | 13.2(9.9-21.8) | 0.842 |
| **Creatinine, μmol/L** | 106(70-243) | 113(71-240) | 99(67-246) | 0.828 |
| **Lactate, mmol/L** | 2.0(1.4-2.8) | 2.0(1.4-2.7) | 2.1(1.4-2.8) | 0.777 |
| **C-reactive protein, mg/L** | 133(69-160) | 160(77-186) | 96(60-159) | 0.007* |
| **Procalcitonin, ng/mL** | 1.8(0.2-15.3) | 2.0(0.4-15.5) | 1.2(0.2-15.1) | 0.375 |
| **Platelets count, ×109/L** | 170(102-227) | 172(105-229) | 165(89-223) | 0.418 |
| **APACHEII score** | 16(12-20) | 16(11-20) | 18(14-24) | 0.009* |
| **SOFA score** | 6(4-9) | 5(4-8) | 8(7-11) | <0.001* |
| **Vasopressors** | 100(49.0) | 51(38.1) | 49(70.0) | <0.001* |
| **NTIS** | 128(62.7) | 78(58.2) | 50(71.4) | 0.064 |
| Data are presented as median (interquartile range), or number (percentage). NTIS, nonthyroidal illness syndrome; APACHE II, Acute Physiology and Chronic Health Evaluation II; SOFA, Sequential Organ Failure Assessment. *p < 0.05. | | | | |

Supplemental Table 2. Baseline characteristics between NTIS and euthyroid patients in the invasive ventilation subgroup.

| **Characteristics** | **Total (n=151)** | **Without Weaning failure (n=97)** | **With Weaning failure (n=54)** | **p value** |
| --- | --- | --- | --- | --- |
| **Age, years** | 69(61-78) | 69(60-78) | 68(63-75) | 0.866 |
| **Sex (male%)** | 110(72.8) | 73(75.3) | 37(68.5) | 0.372 |
| **Comorbidities** |  |  |  |  |
| **Hypertention** | 85(56.3) | 58(59.8) | 27(50.0) | 0.245 |
| **Diabetes** | 48(31.8) | 31(32.0) | 17(31.5) | 0.952 |
| **Chronic kidney diseases** | 31(20.5) | 25(25.8) | 6(11.1) | 0.033* |
| **Infection sites** |  |  |  |  |
| **Respiratory system** | 139(92.1) | 88(90.7) | 51(94.4) | 0.619 |
| **Gastrointestinal tract** | 15(9.9) | 12(12.4) | 3(5.6) | 0.29 |
| **Urogenital tract** | 12(7.9) | 11(11.3) | 1(1.9) | 0.08 |
| **Others** | 13(8.6) | 6(6.2) | 7(13.0) | 0.155 |
| **Laboratory indicators** |  |  |  |  |
| **White blood cell count, ×109/L** | 11.2(8.3-14.9) | 11.1(8.4-15.0) | 11.3(8.2-14.4) | 0.957 |
| **Alanine aminotransferase, IU/L** | 35(15-74) | 33(14-73) | 38(18-74) | 0.086 |
| **Total bilirubin, μmol/L** | 15.2(10.4-22.7) | 15.9(10.1-23.6) | 14.0(10.8-18.8) | 0.409 |
| **Creatinine, μmol/L** | 100(63-212) | 104(67-225) | 95(56-182) | 0.285 |
| **Lactate, mmol/L** | 2.1(1.4-2.8) | 1.9(1.4-2.8) | 2.1(1.5-2.7) | 0.817 |
| **C-reactive protein, mg/L** | 97(44-160) | 100(52-160) | 90(23-198) | 0.429 |
| **Procalcitonin, ng/mL** | 1.9(0.3-16.0) | 1.4(0.2-20.9) | 2.0(0.7-8.9) | 0.358 |
| **Platelets count, ×109/L** | 165(95-221) | 165(91-220) | 166(106-224) | 0.751 |
| **APACHEII score** | 19(15-26) | 19(14-26) | 21(15-26) | 0.399 |
| **SOFA score** | 9(7-11) | 9(7-11) | 9(8-11) | 0.173 |
| **Vasopressors** | 102(67.5) | 61(62.9) | 41(75.9) | 0.101 |
| **NTIS** | 112(74.2) | 69(71.1) | 43(79.6) | 0.253 |
| Data are presented as median (interquartile range), or number (percentage). NTIS, nonthyroidal illness syndrome; APACHE II, Acute Physiology and Chronic Health Evaluation II; SOFA, Sequential Organ Failure Assessment. *p < 0.05. | | | | |
